# Is it reasonable to exclude other severe mental illnesses and mood stabilizers in the prediction of suicide?

**DOI:** 10.1038/s41398-024-02845-8

**Published:** 2024-04-05

**Authors:** Sérgio André de Souza Júnior, Gerardo Autran Cavalcante Araújo, Tainá Rocha Josino, Fábio Gomes de Matos e Souza, Luisa Weber Bisol

**Affiliations:** 1 Federal University of Ceara, Fortaleza, Ceará Brazil; 2 University Hospital Walter Cantidio, Fortaleza, Ceará Brazil

**Keywords:** Bipolar disorder, Scientific community

Dear Editor,

I appreciate the insightful work presented in the article “Predicting suicide risk in 137,112 people with severe mental illness in Finland: external validation of the Oxford Mental Illness and Suicide tool (OxMIS)” by Sariaslan et al. [1]. However, certain methodological considerations warrant attention.

Firstly, the exclusive focus on individuals diagnosed with schizophrenia-spectrum disorder (SCZS) and bipolar disorder (BD) raises questions about the omission of borderline personality disorder (BPD) and major depressive disorder (MDD), both known for elevated suicide risk (45.1% and 19.7%; in contrast with 12.9% of suicide risk to SCZS). A meta-review [2] in which one of the authors was Fazel, highlighted higher suicide risks in these conditions (BPD and MDD), prompting inquiry into their exclusion.

Secondly, the observed threefold higher prevalence of SCZS compared to BD in Finnish sample contradicts epidemiological data that estimate prevalence of SCZS is around 0.32% [3] and BD is 2.4% [4]. An explanation for this discrepancy is crucial for the study’s validity and generalizability.

Thirdly, the exclusion of mood stabilizers from analysis, despite their fundamental role in the treatment of patients with BD, raises concerns. In that paper, the variable “mood stabilizer” was used in the topic “recent medication”. It is difficult to understand why mood stabilizers were withdrawn in the analysis by Sariaslan et al. [1] since 26% of the sample had BD and it is well-recognized that the first-line treatment of BD is a mood stabilizer [5]. Given the documented antisuicidal properties of lithium, its omission necessitates clarification [6].

Fourthly, the disparity in the number of episodes/patients/year between the Swedish [7] and Finnish [1] populations demands explanation. In Sweden, the number of episodes was 574,018 in 75,158 between 2001 and 2008, the number of episodes/patients/years is 0.95. In Finland, the number of episodes was 5,261,732 in 137,112 between 1996 and 2017, the number of episodes/patients/years is 1.74. The 83% increase in the Finnish population suggests potential influencing factors, warranting exploration, such as the role of mood stabilizers.

Lastly, the amalgamation of inpatient and outpatient episodes overlooks the nuanced outcomes associated with each. A more detailed analysis, distinguishing between inpatient and outpatient episodes, would enhance the study’s precision and clinical relevance [8].

In conclusion, while acknowledging the valuable insights provided, addressing these concerns is essential for ensuring the robustness and applicability of the findings. Clarifications and adjustments in subsequent analyses would strengthen the impact of this research on understanding and predicting suicide risk in individuals with severe mental disorders.
